# Resting-State Quantitative Electroencephalography Reveals Increased Neurophysiologic Connectivity in Depression

**DOI:** 10.1371/journal.pone.0032508

**Published:** 2012-02-24

**Authors:** Andrew F. Leuchter, Ian A. Cook, Aimee M. Hunter, Chaochao Cai, Steve Horvath

**Affiliations:** 1 Laboratory of Brain, Behavior, and Pharmacology, Semel Institute for Neuroscience and Human Behavior, University of California Los Angeles, Los Angeles, California, United States of America; 2 Depression Research and Clinic Program, Department of Psychiatry and Biobehavioral Sciences, Semel Institute for Neuroscience and Human Behavior, University of California Los Angeles, Los Angeles, California, United States of America; 3 Department of Human Genetics, David Geffen School of Medicine, Gonda (Goldschmied) Neuroscience and Genetics Research Center, University of California Los Angeles, Los Angeles, California, United States of America; 4 Department of Biostatistics, School of Public Health, University of California Los Angeles, Los Angeles, Caliornia, United States of America; University of Manchester, United Kingdom

## Abstract

Symptoms of Major Depressive Disorder (MDD) are hypothesized to arise from dysfunction in brain networks linking the limbic system and cortical regions. Alterations in brain functional cortical connectivity in resting-state networks have been detected with functional imaging techniques, but neurophysiologic connectivity measures have not been systematically examined. We used weighted network analysis to examine resting state functional connectivity as measured by quantitative electroencephalographic (qEEG) coherence in 121 unmedicated subjects with MDD and 37 healthy controls. Subjects with MDD had significantly higher overall coherence as compared to controls in the delta (0.5–4 Hz), theta (4–8 Hz), alpha (8–12 Hz), and beta (12–20 Hz) frequency bands. The frontopolar region contained the greatest number of “hub nodes” (surface recording locations) with high connectivity. MDD subjects expressed higher theta and alpha coherence primarily in longer distance connections between frontopolar and temporal or parietooccipital regions, and higher beta coherence primarily in connections within and between electrodes overlying the dorsolateral prefrontal cortical (DLPFC) or temporal regions. Nearest centroid analysis indicated that MDD subjects were best characterized by six alpha band connections primarily involving the prefrontal region. The present findings indicate a loss of selectivity in resting functional connectivity in MDD. The overall greater coherence observed in depressed subjects establishes a new context for the interpretation of previous studies showing differences in frontal alpha power and synchrony between subjects with MDD and normal controls. These results can inform the development of qEEG state and trait biomarkers for MDD.

## Introduction

Major Depressive Disorder (MDD) is characterized by dysphoric and anxious mood, difficulties in concentration and decision making, ruminative and self-referential thinking, as well as anhedonia and lack of motivation [1], [2]. These symptoms are consistent with deficits seen in experimental paradigms, in which patients with MDD show deficits in emotional and cognitive information processing [3], [4]. Aberrant emotional processing has been demonstrated in the context of reactions to emotional facial expression or startle in the context of pleasant stimuli [5], [6]. Cognitive deficits have been reported in memory processing, learning, attention, and executive function [7], [8]. While clusters of these symptoms are used to define MDD, their neurobiological origins are not well understood [9]. Elucidating the linkage between the symptoms and pathophysiology of MDD could lead to more accurate and meaningful diagnoses that would have greater prognostic significance [10].

Many of the symptoms and deficits of MDD have been hypothesized to arise from dysfunction in brain networks linking the limbic system and cortical regions [7], [11]. Disruptions in both top-down and bottom-up information processing have been observed with task-activated functional magnetic resonance imaging (fMRI), with altered functional connectivity between dorsolateral prefrontal cortex (DLPFC) and subcortical limbic structures (i.e., amygdala, thalamus) as well as subgenual anterior cingulate cortex [11]–[13]. In addition to task activation studies, resting-state fMRI has been used to examine “resting state networks” (RSNs) that subserve a range of brain processes including executive control, emotional saliency, self-referential information processing, and the default mode network (DMN) [14]–[17]. Studies of the resting state provide an important opportunity to examine connectivity unbiased by any task, and to examine the role that regions may play as parts of multiple networks. Few studies have specifically examined RSNs in MDD. Examination of the resting-state blood oxygen level-dependent (BOLD) signal in MDD shows primarily broad increases in functional connectivity in the DMN and other networks [18]–[21], although other studies have found decreased resting connectivity between some regions [22]–[24] or complex reciprocal relationships between cortical and subcortical structures [25].

Neurophysiologic tools are complementary to fMRI for examining brain network activity. Electroencephalographic (EEG) signals oscillate on a faster time course than BOLD signals [26] with the EEG oscillations actually eliciting the BOLD signal activations within several RSNs [27]. Synchronous EEG oscillations appear to bind together BOLD responses within RSNs in a frequency-dependent manner: long-distance integration of the BOLD response is coordinated by lower frequency (e.g., alpha, or 8–12 Hz) activity, while shorter-distance BOLD responses are coordinated by higher frequency (e.g., beta, or 12–20 Hz) activity [26], [28]–[29]. BOLD signal fluctuations within each RSN are accounted for by different combinations of rhythmic neuronal firing in the delta (0.5–4 Hz), theta (4–8 Hz), alpha, beta, and gamma (>20 Hz) frequency bands, and multiple frequencies are coupled to mediate brain operations [30]–[31]. Each functional network therefore has a distinct electrophysiological signature that is characterized by the synchronous oscillations of the neurons in that network [31]–[32]. In a combined fMRI/qEEG resting state study, Sadaghiani and colleagues showed that spontaneous fMRI fluctuations were strongly positively correlated with alpha band oscillations in a cingulo-insular-thalamic network, and negatively correlated in the dorsal attention network [29]. They concluded that the alpha synchronization plays a key global role in top-down network control, as proposed by Klimesch and colleagues [33].

It has been established that subjects with MDD have dysregulation of neural oscillatory synchrony, but comprehensive information is limited. There is consistent support for increased synchrony in the alpha band, as evidenced by increases within single regions of alpha band power on quantitative electroencephalography (qEEG) [34]–[39]. Studies are inconsistent, however, in identifying which region(s) show this abnormality, with increases reported over the frontal or parietooccipital regions, either on the right or left [39]–[41]. One report found that patterns of alpha asymmetry fluctuated over the span of weeks in subjects with MDD as compared to normal controls [35], suggesting that disturbed synchrony in MDD may reflect a broadly distributed dysregulation [42]–[44]. Disturbed synchrony in other frequency bands has not been consistently reported.

It has been suggested that the disturbed synchrony in neural oscillations may reflect dysfunction within RSNs in subjects with MDD [45]. Most studies of neural synchrony in MDD, however, have examined brain function *within* a single region over a relatively short distance. Few studies have examined synchronous oscillations from sites spanning greater distances, *across* brain regions, to provide information regarding the neurophysiology of larger scale networks [37], [43], [46]–[47]. qEEG coherence is a measure that is well suited to examine synchrony across brain regions. While a peak in qEEG power indicates oscillatory synchrony at a single point, coherence is a well-established indicator of connectivity between two points, or “nodes,” that have a fixed oscillatory phase relationship. Coherence therefore represents the coupling of activity between two nodes that are functionally linked, but not time-locked to a specific event [48]–[50]. Coherence values range between 0 (no shared activity between nodes) and 1 (completely synchronous). This measure thus is well-adapted for assessing functional connectivity in RSNs: it has been successfully used to examine spatial integration both at short- and long-distances in the brain [51], [52], and functional connections among sites overlying disparate cortical areas involved in sensory, motor, and cognitive tasks, both during tasks and at rest [51], [53]–[54]. Coherence was first examined in clinical populations with depression or dementia by O'Connor and colleagues [55], but has not been extensively studied in subjects with MDD compared to healthy controls. The most systematic previous study of connectivity in MDD was conducted by Fingelkurts and colleagues [43], who examined 12 medication-free depressed outpatients and used the index of structural synchrony to analyze nine categories of functional connectivity (e.g., short left/right, short anterior/posterior, long left/right, long anterior/posterior, long interhemispheric) separately for the theta and alpha frequency bands).

**Figure 1:**
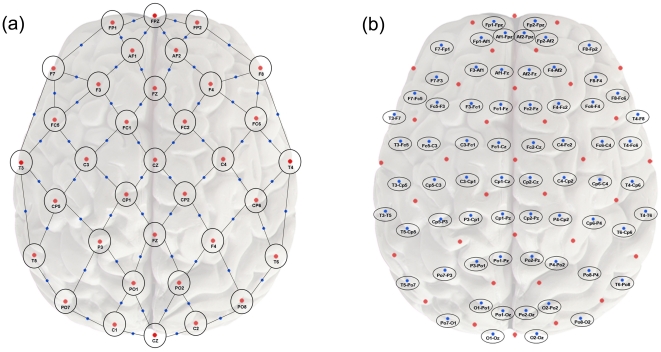
Topographic locations for the electrode montage used in EEG recordings and coherence calculations. Electrode locations were based upon an enhanced version of the International 10–20 System of electrode placement, with additional electrodes placed over the frontal and parietal regions (1A). Locations were projected through Cartesian coordinates onto a two-dimensional representation of the brain, using a central electrode (Cz) as the origin, with locations labeled and indicated by red dots. Recordings were performed referenced to the Pz electrode, and data were recalculated by subtraction offline for a bipolar montage consisting of 66 nearest-neighbor electrode pairs (signified by the lines connecting individual electrodes). Bipolar pairs were considered as nodes of a brain network, with the nodes located at the midpoint between the electrode pairs shown (indicated by the blue dots and the oval labels in 1B). Coherence was calculated between all pairs of nodes as described in the methods.

In the present study, we extend the earlier work of Fingelkurts and colleagues by examining synchrony across brain regions with qEEG coherence using a denser electrode array, a broader range of frequency bands, and a greater number of subjects. We compared the global resting functional connectivity of subjects with MDD and healthy controls utilizing the novel method of weighted network analysis [56], which obviates the need to threshold the observed coherence values. The nodes in the weighted (whole brain) network corresponded to pairs of neighboring qEEG recording electrodes (Figure 1), and the coherence between each pair of nodes was considered as a connection or “edge” of the network. Coherence values representing the strength of the network connections (i.e., value of the edges) were examined in each frequency band to identify any differences in the strength of the resting functional connectivity between groups. To further elucidate patterns of difference in functional connections between MDD and control subjects and characterize brain connectivity in the depressed state, we also examined the mean length of the edges showing significant differences, as well as the locations of the nodes most commonly linked by significant edges.

## Materials and Methods

### Ethics statement

This study was approved by the University of California Los Angeles (UCLA) Office of the Human Research Protection Program. Informed consent was taken via an approved consent form before any study procedures were done. All clinical investigation was conducted according to the principles expressed in the Declaration of Helsinki. Further, all clinical investigations are reviewed in accordance with FDA (Food and Drug Administration) regulations at 21CFR Parts 50 and 56.

### Subjects

This study examined adult subjects ages 21–70 with MDD who had participated in one of four placebo-controlled antidepressant treatment trials conducted over four years in the UCLA Laboratory of Brain, Behavior, and Pharmacology (n = 121) and healthy control subjects who were recruited for a study of the effects of antidepressant medication on normal brain function (n = 37). All depression trials were of similar size, utilized comparable recruitment procedures and inclusion/exclusion criteria, and subjects among the four trials did not differ significantly with respect to age, gender, or symptom severity at intake, so that the data were pooled for these analyses. Healthy control subjects had no current or prior history of any psychiatric or neurologic disorder [57]. All subjects were recruited by community advertisement and were screened for eligibility using a standard clinical evaluation, a structured clinical interview (Structured Clinical Interview for Axis I DSM-IV Disorders – Patient Edition: SCID-I/P, version 2.0) [58], and the 17-item Hamilton Depression Rating Scale (HamD_17_) [59]. Depressed subjects had HamD_17_ scores ≥16 at entry. Exclusion criteria included psychotic symptoms, cluster A or B Axis II disorders, prior suicidal ideation, or any serious medical conditions known to affect brain function or to contraindicate use of the active medication. Subjects were free of psychotropic medications for at least two weeks prior to enrollment. There was no significant difference in the mean age or gender or handedness ratios between the two subject groups, although predictably, the MDD subjects had significantly higher mean HamD_17_ score than did control subjects (Table 1).

**Table 1:** Mean age, gender and handedness ratios, and HamD_17_ scores for MDD and healthy control subjects.

	MDD subjects (n = 121)	Healthy controls (n = 37)	Test Statistic, p-value
Age mean yrs. (SD)	41.5 (12.6)	37.4 (13.4)	t_(156)_ = −1.73, p = .09, N.S.
Gender (F∶M)	75∶46	20∶17	Chi-square = .73, p = .39, N.S.
Handedness (R∶L∶ambi)	99∶21∶1	33∶4∶0	Chi-square = 1.09, p = .58, N.S.
HamD_17_ mean(SD)	21.9 (3.6)	0.70 (0.97)	Student t-test p<E-22

### qEEG recordings

Resting EEG was recorded while subjects lay quietly with eyes closed in a sound attenuated room. Subjects were alerted frequently to avoid drowsiness, and were instructed to remain still and inhibit blinks or eye movements during each recording period. EEG was recorded using a 35-channel enhanced version of the International 10–20 System of Electrode Placement with additional electrodes located over prefrontal and parietooccipital regions (indicated by red dots and labels in Figure 1A). Ag|AgCl electrodes were placed using an electrode cap (ElectroCap, Inc.; Eaton, OH) referenced to Pz. Electrode impedances were balanced and under 5 kΩ for all electrodes. Vertical and horizontal electro-oculograms (EOG) were recorded for identification of eye movement artifact using bipolar electrodes placed at the supraorbital and infraorbital ridge of the right eye and the outer canthi of the left and right eye, respectively.

A minimum of 10 minutes of EEG data were recorded using a 16-bit resolution Neurodata QND system (Neurodata, Inc.; Pasadena, CA) at a sampling rate of 256 Hz, a low-pass filter of 70 Hz, and a high-pass filter of 0.3 Hz, as well as a notch filter at 60 Hz. Data were stored in digital format and imported into Brain Vision Analyzer (BVA) software (Brain Products GmbH; Gilching, Germany) in order to remove offsets, optimize scaling, and re-reference the data through amplitude subtraction into a series of 66 nearest-neighbor bipolar electrode pairs. These pairs are indicated by the lines between electrodes (punctuated by blue dots) in Figure 1A, and are labeled within the ovals in Figure 1B. The data then were segmented into 2-second non-overlapping epochs, and any epochs containing eye movement, muscle, or movement-related artifacts, or amplifier drift were removed using a semiautomated interactive process. Two technologists inspected the data independently using multiple bipolar and referential montages, and isolated and removed data segments containing artifacts. In addition, data were processed using the BVA artifact rejection module that removed data according to standard thresholds likely to represent artifact based upon voltage step gradient (i.e., 100 µV), absolute values of difference within the epoch, and persistent low activity.

### Power and coherence calculations

The power spectral density of the artifact-free bipolar pair EEG data was calculated using the BVA fast Fourier transform (FFT) function. The 512-point FFT was calculated for artifact-free two-second epochs with a rectangular window, DC de-trending applied to each segment of data, and 0.5 Hz overlap at the limits of the band, yielding a frequency resolution of 0.5 Hz. Power was calculated in four frequency bands, corresponding to delta (0.5–4 Hz), theta (4–8 Hz), alpha (8–12 Hz), beta (12–20 Hz), for all nearest neighbor bipolar pairs of electrodes. For the purposes of these analyses, each pair of nearest neighbor electrodes represents a node of a brain network. The 66 nodes were mapped onto a Cartesian projection of the head in a two-dimensional plane, with each node located at the geometric midpoint (indicated by blue dots) between the two individual electrodes (indicated by red dots) in Figure 1B. This mapping allowed calculation of the relative physical distance between nodes in Cartesian coordinate space relative to the origin (in this case, the location of electrode Cz).

qEEG coherence is a measure of the consistency of the phase relationship between two signals and uses surface EEG to make inferences about underlying brain functional connectivity [49]. Coherence was calculated between pairs of nodes, and represents a normalized measure of the functional coupling between the signals at the nodes at any given frequency [60]–[62]. Coherence was calculated as a function of the power spectral outputs for the signals from the separate nodes for each frequency λ:
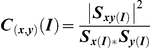
or the square of the cross-spectrum of the two signals *x* and *y* divided by the product of the spectra of the individual channels, at the frequency λ. This procedure yields a real number between 0 (no coherence) and 1 (maximal coherence). Coherence values from individual bins within a frequency band were averaged to obtain the coherence value for that band.

### Data analysis

#### Weighted network analysis

The data were analyzed according to the principles of weighted network analysis (WGCNA) using the methods implemented in the WGCNA R package [56], [63]. Weighted networks preserve the continuous nature of the underlying coherence information and do not require one to choose a threshold value. For the network analyses performed here, the relative locations of the electrodes were mapped in Cartesian coordinate space with electrode Cz at the origin, and each electrode's coordinates specified as *T_x,y_* relative to the origin. Nodes were formed from pairs of adjacent electrodes, such that the 35 individual electrodes yielded 66 separate nodes. Nodes *N_X,Y_* were specified as located at the midpoint between the two electrodes *T_X,Y_* and *T_X′,Y′_* comprising the node (Figure 1), with the locations *N_X_* and *N_Y_* calculated as:
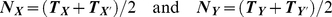
Each pair of nodes was considered a connection or network “edge” 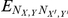, where *N_X,Y_* and *N_X′,Y′_* represent the two nodes whose connection comprised the edges, for a total of 2,145 edges between each pair of nodes in each frequency band. Each edge was characterized by two complementary measures: 1) connection strength 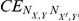, which is the coherence value between the two nodes; and, 2) connection length 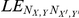, which represents the physical distance between the two nodes comprising the connection. Lengths were calculated from the Cartesian coordinate map (Figure 1) according to the formula
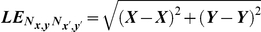
Finally, the degree of each node (also known as overall node connectivity) was calculated, defined as the average of the coherence values for a node with the other 65 nodes according to the formula
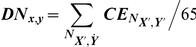
The median values of connection strengths (***CE***) were compared for the two groups in each frequency band using the Kruskal Wallis test. The median length values (***LE***) of those connections that showed differences in strength also were compared for the two groups in each frequency band using the Kruskal Wallis test. To identify MDD related hub nodes, Student's t-test was used to test whether the mean node degree (***DN***) in MDD subjects differed from that in controls. A strict Bonferroni correction of 2.33×10^−5^ (0.05/2,145) was imposed on all analyses involving network connections, and of 7.57×10^−4^ (0.05/66) was applied to all analyses involving hub nodes, to protect against false positive findings. Locations of significant connections and hub nodes were tabulated. Associations between significant edges, hub nodes, and severity of depression (as measured by HamD_17_ scores) and by age were examined using Pearson correlations. Differences in edge and hub node values by gender were examined using the Kruskal Wallis test. In addition, other tests for statistical significance (e.g., the Kruskal Wallis test p-value and the q-value) were performed and are reported as supplementary data (Table S1).

The nearest centroid analysis method as implemented in the WGCNA R library [63], was used to determine which combination of edges best characterized MDD subjects and differentiated them from normal controls. Because the subject pool was unbalanced with regard to group size (121 MDD versus 37 control subjects), the MDD subjects were divided into four datasets reflecting their original study source (groups of 31, 29, 25, and 36 subjects), such that each of the datasets contained roughly the same number of subjects and was comparable to the number of control subjects. The final nearest centroid categorization used the rankings from the four separate datasets and combined them with the metaanalysis method implemented in the rankPvalue function (pValueLowScale) of the WGCNA R library. Within each of the four datasets, we performed five-fold cross validation in which the data were split into five bins, with four of these used at any one time as a training set and the remaining bin used as a test set. Edge connectivity selection (based on the correlation test) in each of the training sets was performed separately in order to avoid biasing the results. Thus, each of the four datasets led to cross-validated estimates of the classification accuracy (percentage of subjects correctly classified). The four cross-validated estimates were averaged to arrive at a final unbiased estimate of the classification accuracy. Results of supervised clustering based on the most significant edges that defined the nearest centroid predictor of group membership were displayed in a hierarchical cluster tree map.

## Results

**Figure 2:**
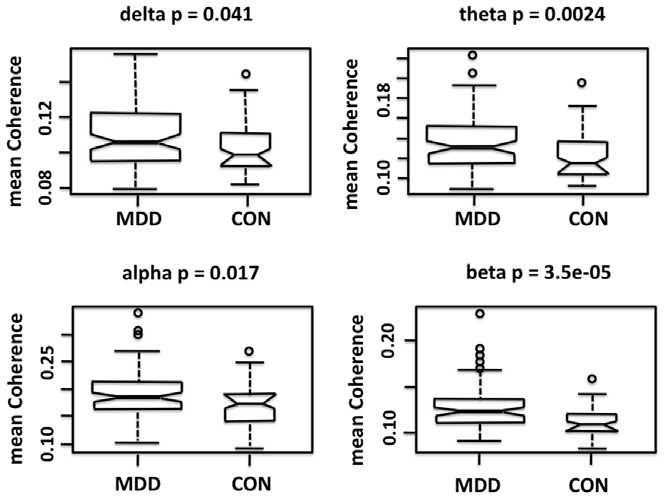
Boxplots of median coherence for MDD and healthy control groups (by frequency band). The short horizontal line within each box shows the median values, and the notches represent 95% confidence intervals for the median values. Statistical significance listed for each frequency band is based upon the Kruskal Wallis test.

**Figure 3:**
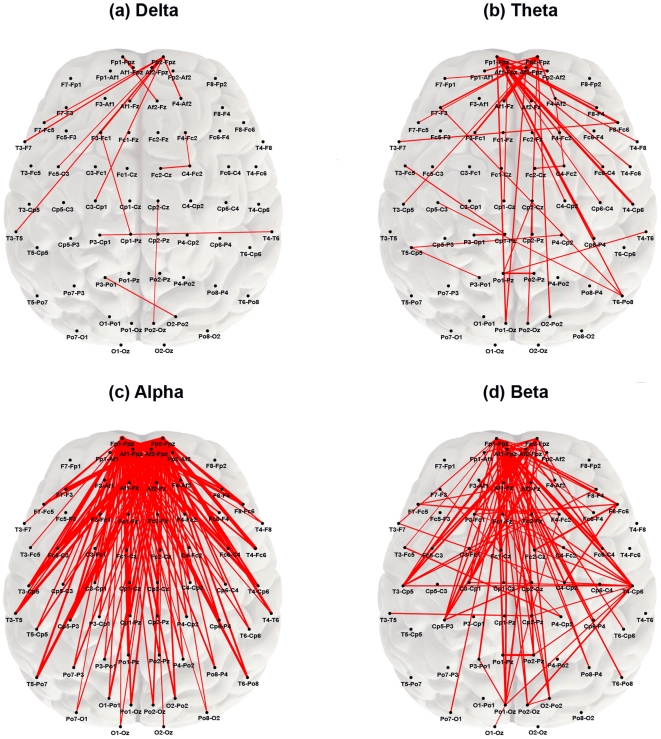
Map of connection strengths 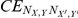
 **showing significant differences between groups (by frequency band).** Red lines represent connections (edges) whose strength remained significantly different between MDD and control subjects after Bonferroni correction (p≤2.33×10^−5^). All red edges represent coherence values that were greater in the MDD group with line thickness proportional to the magnitude of the difference. The nodes most commonly involved in significant edges across frequency bands were located in the prefrontal region.

MDD subjects showed statistically significantly greater connection strength ***CE*** (higher overall median coherence across all edges) than controls in each of the four frequency bands, but most notably in the beta band (Kruskal Wallis p = 0.000035) (Figure 2). The topography of individual connections (edges) showing significantly greater strength ***CE*** in MDD subjects is displayed by frequency band in Figures 3A–D. In the delta and theta bands, relatively few highly significant differences in connection strength were found after applying Bonferroni correction. The most significant differences in ***CE*** in the delta band (17 edges) and theta band (42 edges) were seen between the frontopolar and temporal regions. In the theta band, highly significant edges also were found between the frontopolar and parietooccipital regions, and between the temporal regions bilaterally (Figures 3A–B). The alpha band contained the greatest number of significantly different connections (141 edges) and these linked brain regions that were more widely separated, including connections between the frontopolar or DLPFC and the temporal or parietooccipital regions bilaterally (Figure 3C). The beta band also contained a large number of significantly different connections (121 edges) that formed a dense network within the frontal and temporal regions, both within and across hemispheres. There were fewer differences in long distance connections between the prefrontal and posterior regions in this band compared with lower frequency bands (Figure 3D). Further detail on the differences in connection strength ***CE*** between MDD and control subjects, with results for each edge in each frequency band, are shown in Table S1. This Table S1 reports details of Student's-t and Kruskal Wallis tests, associated p-values, and q-values. For all significant edges, the mean coherence values were higher in the MDD than in the control group.

**Figure 4:**
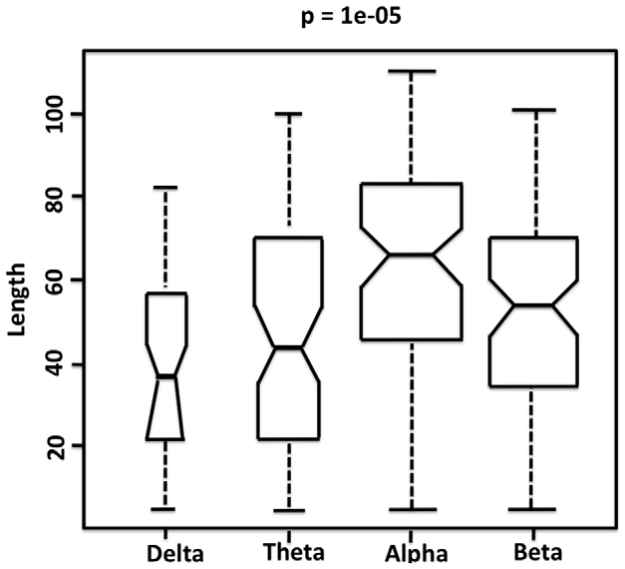
Boxplots of edge lengths 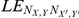
 **of connections that showed significant difference between groups (by frequency band).** Edge length was determined from the relative physical distance between nodes on a two-dimensional plane as shown in Figure 1B. Edges with significantly different connection strength differed significantly in length across frequency bands (p = 0.00001). Significance level represents the p value for the Kruskal Wallis test examining the equality of the median edge length values between groups. Short horizontal lines within boxes show the median edge length, with notches indicating 95% confidence intervals of the medians. Median edge length was significantly greater for alpha than any other band. The width of the bars is proportional to the number of edges that were significantly different between groups in the frequency band: in the delta band, there were 17 significant edges; in theta, 42; in alpha, 141; and in beta, 121.

The median physical length ***LE*** of those connections that showed differences in strength also differed significantly across frequency bands (p = 0.00001) (Figure 4). Edge length ***LE*** was significantly greater in alpha than in any other band, and beta length was significantly greater than that for the delta band.

**Figure 5:**
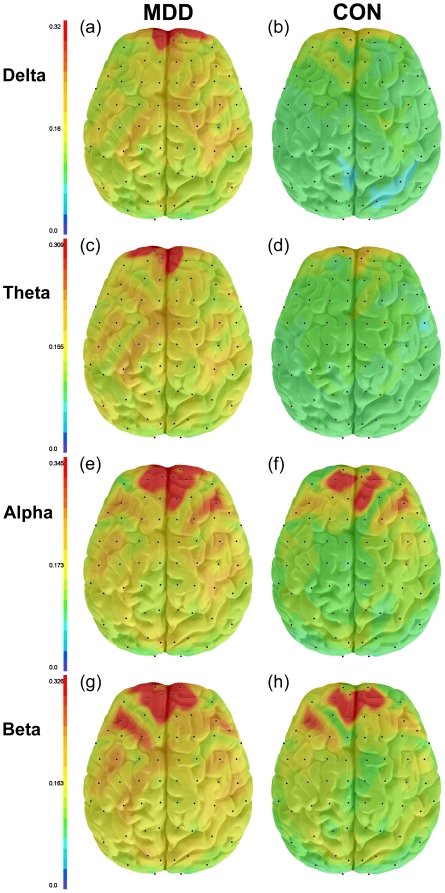
Maps showing the median connectivity *CE* (coherence) between hub node Fp1-Fpz and all other nodes in all frequency bands, separately for MDD and healthy control subjects. This node demonstrates broadly higher median connectivity in the MDD subjects (A, C, E, and G) compared to the control subjects (B, D, F, and H). Coherence values are indicated by the color bar on the left of the maps. Coherence values decrease with distance from the hub node in both MDD and control subjects, but show greater decrease with distance in control subjects.

A number of nodes were identified as hub nodes that had significantly different degree ***DN*** (i.e., average coherence with all other nodes) between the MDD group and normal controls (Table 2). Two frontopolar hub nodes, Fp1-Fpz and Fp2-Fpz, met the Bonferroni threshold for significance in each of the four frequency bands. In the theta and alpha band, these same nodes plus two DLPFC nodes, Af1-Fpz and Af2-Fpz, had significantly higher degree ***DN*** in subjects with MDD. These four nodes showed broadly higher connectivity ***CE*** in the alpha band with all brain regions in MDD subjects compared with healthy controls. In the beta band, these same four plus 21 additional hub nodes showed greater connectivity in MDD than in controls. Across all four frequency bands, no hub node had higher connectivity in controls than in MDD subjects. Maps showing the median connectivity ***CE*** between one of these nodes, Fp1-Fpz, and all other nodes in all frequency bands for MDD and control subjects separately are presented in Figure 5.

**Table 2:** Mean node connectivity ***DN*** (degree) for hub nodes for MDD and control subjects.

Node	p	MDD Mean (± SE)	Normal Mean (± SE)
***Delta***			
Fp1-Fpz	0.00021	0.1 (0.0022)	0.085 (0.0026)
Fp2-Fpz	0.00034	0.1 (0.0022)	0.085 (0.0024)
***Theta***			
Fp1-Fpz	7.90E-06	0.12 (0.0031)	0.091 (0.0036)
Af1-Fpz	5.00E-04	0.15 (0.0034)	0.13 (0.0055)
Fp2-Fpz	2.30E-05	0.12 (0.0031)	0.091 (0.0033)
Af2-Fpz	5.90E-05	0.15 (0.0033)	0.13 (0.0048)
***Alpha***			
Fp1-Fpz	5.40E-08	0.16 (0.0062)	0.097 (0.0029)
Af1-Fpz	4.50E-06	0.2 (0.005)	0.15 (0.0073)
Fp2-Fpz	1.20E-08	0.16 (0.0059)	0.097 (0.0031)
Af2-Fpz	9.30E-06	0.2 (0.0054)	0.15 (0.0063)
***Beta***			
Fp1-Fpz	1.50E-06	0.11 (0.0028)	0.087 (0.0027)
Af1-Fpz	3.30E-07	0.14 (0.0028)	0.11 (0.0033)
F3-Af1	1.00E-04	0.11 (0.0023)	0.095 (0.0026)
F7-Fc5	2.80E-04	0.12 (0.0024)	0.1 (0.0032)
F3-Fc1	2.40E-04	0.13 (0.0026)	0.11 (0.0041)
Fc1-Fz	4.00E-04	0.12 (0.0027)	0.11 (0.0032)
T3-Fc5	6.50E-04	0.13 (0.0024)	0.11 (0.0029)
T3-Cp5	7.30E-05	0.13 (0.0026)	0.11 (0.0029)
Cp5-C3	3.60E-04	0.14 (0.0026)	0.12 (0.0035)
Po1-Pz	7.80E-05	0.14 (0.0026)	0.12 (0.0039)
Po1-Oz	4.60E-50	0.14 (0.0024)	0.12 (0.0035)
Fp2-Fpz	8.70E-06	0.11 (0.0026)	0.088 (0.0024)
Af2-Fpz	4.30E-06	0.13 (0.0026)	0.11 (0.0036)
F8-F4	6.70E-04	0.13 (0.0025)	0.12 (0.0037)
Af2-Fz	1.50E-04	0.13 (0.0025)	0.11 (0.0032)
F8-Fc6	7.10E-05	0.12 (0.0023)	0.1 (0.0031)
F4f-C2	1.70E-04	0.13 (0.0025)	0.11 (0.0038)
Fc6-C4	3.00E-04	0.13 (0.0025)	0.12 (0.0032)
Fc2-Cz	6.80E-04	0.13 (0.0028)	0.11 (0.0034)
T4-Cp6	1.20E-04	0.13 (0.0026)	0.11 (0.0026)
C4-Cp2	3.90E-04	0.13 (0.0024)	0.12 (0.0035)
O2-Po2	5.00E-04	0.13 (0.0024)	0.12 (0.0038)

Hub nodes are those that had significantly different average coherence with all other nodes between the two groups after Bonferroni correction. For each of these nodes, connectivity was greater in the MDD subjects.

**Figure 6:**
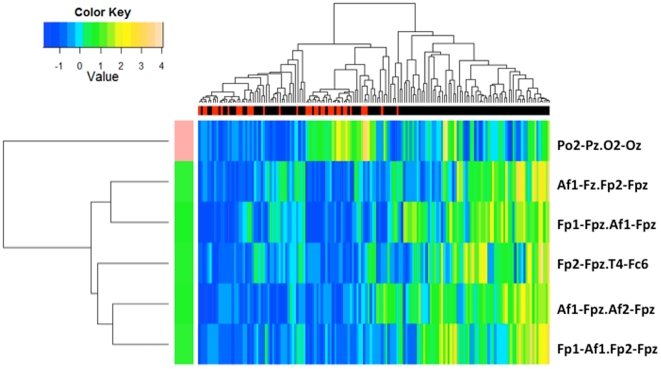
Nearest centroid classification of MDD and healthy control subjects. Six edges (listed on the right) selected using nearest centroid analysis classified subjects into MDD and control groups, with classification indicated by the dendrogram at the top of the figure. Individual subjects are represented by the terminal branches of the dendrogram, with MDD subjects clustering toward the right (indicated by black bars in the top row) and control subjects clustering toward the left (indicated by red bars) in the supervised cluster analysis. Data values for each subject are indicated by a color column in the heatmap corresponding to a terminal branch. MDD subjects tended to have higher coherence values than controls on edges involving frontopolar electrodes, while controls tended to have higher coherence on the edge involving parietooccipital electrodes (indicated by green-to-yellow colors in the heatmap). As part of the clustering algorithm, the coherence values were scaled to have zero mean and unit variance across the subjects (as shown in colorbar).

The nearest centroid classification analysis identified six edges in the alpha band that best characterized the depressed state. Five of these edges involved a hub node, with higher coherence values in the MDD group: three of these edges connected pairs of nodes between the left and right frontopolar and DLPFC regions (Af1-Fz and Fp2-Fpz, Af1-Fpz and Af2-Fpz, and Fp1-Af1 and Fp2-Fpz), one connected a pair between the left frontopolar and DLPFC regions (Fp1-Fpz and Af1-Fpz), and one a pair between the right frontopolar and right temporal regions (Fp2-Fpz and T4-Fc6). The sixth edge connected a pair of nodes in the right parietooccipital region which were not hub nodes, and for which coherence was lower in the MDD group than controls (Po2-Pz and O2-Oz). Supervised hierarchical clustering (Figure 6) shows that MDD cases (shown in black in the top color bar) tend to cluster together, indicating that the combinations of these edges discriminate cases from controls. The cluster tree on the left side shows the relationship among the edges. The bottom five edges (indicated by green in the left color bar) are over-expressed in cases and are positively correlated with each other. The sixth edge (Po2-Pz and O2-Oz) is anti-correlated with the other five edges. This classifier was cross-validated across the four sets of subjects, corresponding to the four datasets that were pooled for this study. On average, the classifier accurately distinguished 81% of MDD from healthy control subjects.

There was no significant difference between the mean number or values of significant edges or hub nodes between male and female MDD subjects. There also was no significant association between mean edge or hub node value and severity of depression as measured by the 17-item Hamilton Depression Rating Scale, or between edge or hub node value and age (data not presented).

## Discussion

These results indicate that subjects with MDD differ significantly from healthy control subjects in patterns of brain functional connectivity. A large number of highly significant edges, in all frequency bands, showed higher functional connectivity in MDD as compared to controls. These differences were most notable in the alpha and beta bands. The hub nodes most often involved in increased connectivity were located in the frontopolar and DLPFC regions, although the patterns of connectivity involving these nodes differed by frequency: in the alpha band, these nodes were involved in significantly longer distance edges than in the beta band. Examination of the most significant edges in the alpha band showed that the connections were *between* the frontopolar or DLPFC regions and the temporal or parietooccipital regions, whereas in the beta band, the connections were most often *within* the prefrontal, temporal, or less often the parietooccipital regions. Nearest centroid analysis indicated that six connections in the alpha band, five of which showed higher connectivity between the frontopolar and DLPFC or frontopolar and temporal regions, and one of which showed lower connectivity within the parietooccipital region, differentiated MDD from control subjects with 81% accuracy.

The patterns of difference between MDD and control subjects, which are consistent with earlier results from Fingelkurts and colleagues [43], should be interpreted within the context of prior research regarding the role of rhythmic oscillations in regulating brain activity. Rhythmic activity overall helps to bind cell assemblies together into functional units: lower frequency oscillations (in the alpha and theta range) operate at a broader level across the brain, binding more distant areas into functional units through “top-down” control, and modulating the activity of local functional units that are bound together by faster oscillations [33], [64]–[65]. The present findings are consistent with this functional topography of alpha and beta oscillations in the brain. Increased alpha coherence was observed in edges that span relatively greater distances (e.g., between prefrontal nodes and more distant temporal or parietooccipital regions), whereas increased beta coherence was evidenced in shorter distance edges (e.g., within frontal or temporal regions).

These findings, which suggest a broad loss of selectivity in functional connections in MDD, are consistent with the reports of Sheline and colleagues [20] as well the Zhou [21] and Greicius [18] groups, which showed significant increases in resting-state cortical functional connectivity in MDD using fMRI. The location of the prefrontal hub nodes that showed the most frequent involvement in increased coherence in the present study approximately coincides with the dorsomedial prefrontal cortical area found by Sheline's group to constitute a “dorsal nexus” of increased connectivity [20]. The fact that the most significant increases in coherence were found in the alpha frequency band could be interpreted as a failure of the top-down control exerted by rhythmic alpha activity. This rhythm is generated by the cortex under the influence of corticothalamic neuronal loops [66]. Greicius and colleagues showed significantly increased thalamic functional connectivity with the default mode network at rest in MDD, supporting the concept of dysfunction in the top-down control circuit that is mediated by rhythmic alpha activity [18]. The increases in longer-distance alpha coherence could in turn mediate the local increases seen in beta coherence; there is significant cross-frequency interaction, such that top-down alpha band oscillatory processes and bottom-up high frequency oscillatory processes may be functionally coupled [67]. The possibility that increased alpha coherence may in part be the result of a bottom-up input from local processes in the beta frequency band, however, cannot be ruled out.

The present findings establish a new context for interpretation of previous studies showing differences in frontal alpha band power and synchrony between subjects with MDD and normal controls [34]–[36], [38]. Studies have shown increases in synchronized frontal alpha activity and qEEG alpha power, although the lateralization has varied, with relatively greater alpha power reported both over left and right anterior regions [34], [36], [68]. It is possible that shifting power asymmetries previously reported [35] may reflect the effects of significantly increased functional connectivity in subjects with MDD. Recent results indicate that interhemispheric interactions are related to shifting lateralization on a moment-to-moment basis in MDD [69]. Future studies should examine the role of increased connectivity in modulating asymmetries in frontal power.

Few previous studies have assessed resting state functional connectivity in MDD. Winterer and colleagues reported that depressed alcoholic patients had significant increases in coherence in the alpha and beta bands in the posterior regions, although alcoholics without depression did not [70]. Fingelkurts and colleagues examined the “index of structural synchrony,” a different measure of signal synchronization, and found that subjects with MDD had broad significant increases in alpha and theta band functional connectivity [43]. These differences consisted primarily of increased short distance functional connections in the left and long-range connections in the right hemisphere. They interpreted these increases as adaptive and compensatory mechanisms aimed at overcoming deficient semantic integration. Hinrikus and colleagues found that depressed subjects had increased coherence between some brain regions, but examined only interhemispheric coherence between small numbers of locations and detected no statistically significant difference [71]. Other studies of coherence have used methods that differ from the current study, and have obtained disparate results. Knott and colleagues [37] found decreased coherence in MDD subjects compared to normal controls, but calculated coherence between a limited number of individual electrodes, a technique that may not characterize regional measures of brain activity as well as the electrode pairs in the present study [61]. Armitage and colleagues have examined coherence during sleep and shown that it is decreased among adolescents with MDD, and is a predictor of recurrence and risk of developing illness [72]–[74]. The relationship between sleep and resting awake state coherence is unknown.

Greicius and colleagues speculated that the increased functional connectivity in mood regulating networks might be associated with impaired cognitive processing in MDD [18]. This speculation is consistent with the established role of oscillatory activity in regulating cognitive networks [32], [75]. The ability to modulate alpha rhythmicity and coherence has been linked to the ability to shift and focus attention, and meet working memory and executive demands [51], [53]–[54], [76]. Successful modulation of beta activity has been related to response preparation and cognitive control [77]–[78]; “pathological” increases in beta activity are associated with deterioration in cognitive flexibility and control [78]. Several neurophysiologic measures of synchronization, including coherence, phase synchronization, and synchronization likelihood, have been related to deficits on measures of attention and working memory, as well as processing of auditory, visual, linguistic, and social cognition information in psychiatric and neurologic illnesses [75]. This wide range of cognitive activities overlaps with the cognitive domains and functions that have been reported to be deficient in some subjects with MDD [3], [79]. Theta oscillations play a significant role in memory function, with modulated coupling of theta oscillations between the prefrontal, parietal, and temporal cortices prominently involved in memory encoding and recall [80]–[82]. In the present study, those edges showing significantly increased coherence in the theta band involved connections between prefrontal and temporal regions. These connections may have special functional significance related to memory dysfunction in MDD, and should be explored in future studies.

Experimental data also link synchronization of neuronal oscillations to the ability to process emotional information. Kostandov and colleagues reported that processing of the emotional content of facial expression was associated with increases in coherence in the theta and alpha frequency ranges, particularly involving the dorsolateral frontal and temporal cortices [83]. Similarly, Balconi and colleagues found that processing of positive and negative visual images, or masked emotional facial expressions [84], was associated with increases in coherence in the delta, theta, and alpha bands, depending on the nature of the task and stimulus, and particularly from the frontal regions. In addition to processing of emotional content, the subject's internal emotional state may be mediated by the degree of synchronization. Andersen and colleagues reported that anxious rumination in healthy volunteers was associated with increases in theta and alpha band coherence [80]. This finding is consistent with the results reported here that MDD is associated with an increase in theta and alpha coherence, and also is consistent with Greicius' speculation that increased connectivity associated with MDD may operate to the detriment of other types of brain processing [18]. If networks are saturated with the load of processing emotional information, there may be limited capacity to modulate synchronization in response to other processing demands.

Previous reports have highlighted disruption of brain regulatory mechanisms in MDD, focusing on “hubs” of the mood regulatory network such as the rostral anterior cingulate (rACC) [85] or the dorsal nexus posited by Sheline and colleagues [20]. Disruption of normal connectivity patterns could explain many of the regulatory, cognitive, neurovegetative, and emotional symptoms of MDD [75], [86]–[87]. It remains unclear what fundamental mechanism underlies and perpetuates network dysregulation. The current results are consistent with a growing body of literature implicating disturbed brain oscillatory activity in the pathogenesis of MDD [42], [88]–[90]. Modulation of cerebral oscillatory activity plays a central role in regulation of mood, and processing of affective information and emotional stimuli [91]–[93]. Interestingly, synchronization of oscillatory activity is strongly influenced by central serotonergic tone [89]. Serotonergic projections from the medial septal area inhibit hippocampal theta oscillatory synchrony [94], while alpha synchrony is modulated by serotonergic projections from the raphe nuclei to the intralaminar and medial thalamic nuclei [95]. Furthermore, oscillatory activity and related behaviors are modulated by administration of antidepressant medication in animals [94], [96]–[97]. Oscillatory synchrony could represent the neurophysiologic link between neurochemical activity and brain network functions that regulate mood, affect, and processing of emotional information. Oscillatory dysregulation may similarly represent the pathophysiologic link between disturbances in monoaminergic neurotransmission and brain network dysfunction in MDD. Future research should more closely examine the regulation of oscillatory synchrony in subjects at risk for or recovering from MDD, as well as the effect of antidepressant treatments on oscillatory synchrony in MDD.

There are several limitations to the current study. First, limited information was available on the specific symptoms of the MDD subjects and the number of prior episodes they may have had, so we cannot relate the increased connectivity to specific subtypes of the illness. Second, because all subjects in the present study either were experiencing a current major depressive episode or were healthy controls, it is unclear whether elevated connectivity would resolve with treatment or it would be a persistent trait marker for those with a predisposition to the illness. Third, in this study we examined only a single measure of neurophysiologic connectivity, coherence, which indicates the linear association between time-series curves in a frequency band [60]. Absence of a statistical association between two processes does not necessarily exclude a physiologic connection [98]–[99]; conversely, presence of an association does not necessarily indicate a physiologic connection, as EEG signals show a finite correlation even when recorded from separate subjects (secondary to the finite epoch time and similar bandwidth of signal pairs) [100]. Finally, although there is strong evidence of correspondence between surface EEG and brain functional activity in underlying structures [26]–[29], EEG coherence, like any metric derived from electrical recordings from the scalp does not directly measure brain activity. Connectivity of brain regions is inferred from electrical activity recorded at surface sites overlying the various cortical regions.

There is no single technique that has proven to be ideal to study the interaction between two brain signals from scalp recordings. Coherence measures are susceptible to both volume conduction and electrode reference effects [101]–[103], although in the present study both effects were minimized through calculating coherence from closely spaced bipolar electrode pairs [102]. This strategy renders these confounding influences negligible for close bipolar pairs separated from one another by more than 4–5 cm [104]–[106], although volume conduction still may increase coherence for shorter distances depending upon the frequency band and the orientation of the dipole source [103]. It is highly unlikely, however, that any of the differences reported between the MDD and healthy control groups in the current study would arise from volume conduction or reference effects because the electrode montage and recording techniques were identical for both depressed and control groups. Nevertheless, future studies also should consider use of surface Laplacian [107] and Independent Component Analysis (ICA) [108]–[109] EEG methods, as well as phase synchrony [110] connectivity measures, that may help further minimize the effects of volume conduction. Use of high-density electrode arrays in future studies also would help to define more clearly the brain regions showing differences in brain connectivity between MDD and control subjects.

These findings indicate that resting state neurophysiologic connectivity is increased broadly across all brain regions in MDD. Future studies also should more closely examine clinical features of subjects with MDD, including cognitive profiles, functional status, and response to treatment in relation to connectivity measures, in order to determine the possible role of increased functional connectivity as a diagnostic or prognostic marker for MDD.

## Supporting Information

Table S1 **Supplementary data on mean differences in connection strength between MDD and control subjects across frequency bands.** The rows of the table correspond to the connections (edges) between nodes. For each frequency band, the columns report the following measures: the Pearson correlation between edge coherence and MDD status; the Student t-test statistic and p-value; the fold change defined as mean value in MDD cases divided by the mean value in controls; the mean value in the first group (i.e., MDD cases) and the corresponding standard error; and, the value for the Kruskal Wallis test, which is a non-parametric group comparison test that does not assume normality. The q-value represents the expected False Discovery Rate (FDR), controlled with the local FDR method/algorithm [111].
(XLS)
